# Bisexual Attract-and-Kill: A Novel Component of Resistance Management for Transgenic Cotton in Australia

**DOI:** 10.1093/jee/toac032

**Published:** 2022-04-14

**Authors:** Peter C Gregg, Alice P Del Socorro, Sarah Wilson, Kristen M Knight, Matthew R Binns, Philip Armytage

**Affiliations:** School of Environmental & Rural Science, University of New England, Armidale, NSW 2350, Australia; School of Environmental & Rural Science, University of New England, Armidale, NSW 2350, Australia; Formerly AgBiTech Australia, PO Box 18281, Clifford Gardens, Toowoomba, QLD 4350, Australia; Bayer Crop Science, PO Box 92, Harlaxton, QLD 4350, Australia; Department of Agriculture, Water and the Environment, GPO Box 858, Canberra, ACT 2601, Australia; AgBiTech Australia, PO Box 18281, Clifford Gardens, Toowoomba, QLD 4350, Australia

**Keywords:** *Helicoverpa armigera*, *Helicoverpa punctigera*, attract-and-kill, transgenic cotton, resistance management

## Abstract

In Australia, destruction of overwintering pupae of *Helicoverpa armigera* (Hübner) and *H*elicoverpa *punctigera* (Wallengren) (Lepidoptera: Noctuidae) has been a key component of mandatory resistance management schemes to constrain development of resistance to Bt toxins in transgenic cotton. This has been accomplished by tillage (‘pupae busting’), but it is expensive and can interfere with farming operations. Bisexual attract-and-kill technology based on plant volatile formulations offers a potential alternative in some circumstances. We discuss strategies for using such products and describe two trials in which three applications of an attract-and-kill formulation substantially reduced the numbers of *Helicoverpa* spp. moths and the numbers of potentially overwintering eggs they laid. One trial tested a curative strategy in which the last generation of moths emerging from transgenic cotton was targeted. The other tested a preventive strategy which aimed to reduce the numbers of eggs in the last generation. The preventive strategy reduced egg numbers by about 90% and is now included as an optional alternative to pupae busting in resistance management strategies for Australian cotton. It is limited to fields which have not been defoliated prior to 31 March and was developed to be used primarily in southern New South Wales. In the 2020–2021 cotton season, it was adopted on approximately 60% of the eligible cotton area. We describe the process whereby the strategy was developed in collaboration with the transgenic technology provider, supported by the cotton industry, and approved by the regulatory authority.

The noctuid moths *Helicoverpa armigera* (Hübner) and *Helicoverpa punctigera* (Wallengren) were major pests of cotton in Australia prior to the widespread adoption of transgenic Bt cotton. Their management typically required 10–15 applications of insecticides per season (Fitt 1989, 2000). Following the introduction of transgenic cotton varieties expressing toxins from *Bacillus thuringiensis* Berliner, along with other integrated pest management tactics, insecticide use against *Helicoverpa* spp. was reduced to an average of less than one spray per season (Wilson et al. 2013, 2018). However, *H. armigera* has an extensive history of resistance to pesticides in Australia (Forrester et al. 1993) and elsewhere (McCaffery 1998) and was considered to pose a significant risk for resistance also to Bt in Australia. *H. punctigera* exhibited much lower levels of resistance to insecticides (Gunning and Easton 1994) and was initially considered to pose less risk, though transient increases in resistance frequency (Downes et al. 2010a) forced a revision of this assessment.

The Australian cotton industry is now dominated (>95%) by Bollgard (Bayer Crop Science, Melbourne, Australia) 3 varieties expressing Cry1Ac, Cry2Ab, and Vip3a toxins. To ameliorate resistance, the industry has adopted a proactive, integrated resistance management scheme for Bt cotton (Downes et al. 2010b, Wilson et al. 2013, Cotton Australia 2021). Key elements of the scheme include planting refuge crops (non-Bt cotton or pigeon peas); (Baker and Tann 2014, Baker et al. 2016a), adhering to planting windows (Baker et al. 2016b), and using postharvest crop destruction followed by tillage to destroy overwintering pupae (‘pupae busting’; Sequeira and Playford 2001, Wilson et al. 2013).

Adherence to the resistance management strategy is mandatory for farmers, and compliance is monitored through audits by the technology provider, Monsanto (now Bayer). Failure to comply can result in retrospective resistance mitigation strategies, including, but not limited to, the requirement to plant additional refuge areas in the following season. While compliance levels are generally high, one requirement that frequently poses difficulties is pupae busting. The current version of the resistance management scheme (Cotton Australia 2021) requires that crops not defoliated before 31 March must be pupae busted by 31 July. This involves cultivation to a depth of at least 10 cm, and to a width of 30 cm on either side of the plant line, in order to disrupt the emergence tunnels of overwintering pupae. Pupae busting can produce high mortality (Wilson et al. 1979) and is therefore valuable in resistance management. However, cultivation is expensive and can be associated with agronomic difficulties such as damaging soil structure and restricting crop rotation options. It is often interrupted by rainfall, especially in southern New South Wales where winter rain is more frequent, making compliance by the due date problematic.

Bisexual attract-and-kill techniques (Gregg et al. 2018) offer potential alternatives to pupae busting. Magnet is a formulation based on research in Australia through successive Cotton Cooperative Research Centers (Del Socorro et al. 2010a, b; Gregg et al. 2010a, b; Mensah et al. 2013) and was registered and commercialized by AgBiTech Australia (Gregg et al. 2010b, Gregg et al. 2016a) for use against *H. armigera* and *H. punctigera*. It comprises a mixture of plant volatile compounds and sugar as a feeding stimulant. Farmers add one of three approved insecticides before application (Del Socorro et al. 2010b). The formulation is applied either from the ground or by air, and in bands which cover 1–2% of the field with coarse droplets (1–5 mm) that moths ingest. Both male and female moths are attracted to and feed on these droplets, resulting in high levels of mortality, and substantial reductions in oviposition (Mensah et al. 2013, Gregg et al. 2016a).

Conceptually, there are three ways in which Magnet and similar bisexual attractants might be used in resistance management, especially for *H. armigera*:

1. Preventive management, in which the generation that lays the eggs that will develop into overwintering pupae is targeted. In most Australian cotton areas, this generation typically occurs in February. The aim is to reduce the immature population so the abundance of subsequent overwintering pupae is reduced to similar levels that can be obtained after pupae busting.
2. Curative management, in which the last, prediapause generation of moths is targeted. Overwintering diapause in *H. armigera* is progressively induced in larvae pupating between late March and mid-April (Wilson et al. 1979, Baker et al. 2016a). Moths emerging from pupae which formed in early to mid-March do not return to and oviposit on cotton, which by this time has matured and may even be defoliated. Instead, they fly to other host plants, both cultivated and wild, taking resistance alleles with them. Killing these moths by treating late season cotton has the potential to prevent the dissemination of resistance and reduce regional resistance frequencies.
3. Remedial management, in which moths are targeted in spring, when they emerge from overwintering diapause. This typically occurs in early to mid-October, before cotton has been planted, so treatment would need to be applied on the old crop residue or on nearby alternative crops such as wheat. This tactic might be used to remediate fields which had not had adequate pupae busting (e.g., because of rain) by the due date.

In *H. punctigera*, diapause is much weaker (Cullen and Browning 1978), fewer overwintering pupae are present particularly in southern cotton regions (Duffield 2004), and the species is considered to be more migratory, especially from noncropping regions (Gregg et al. 2019). Hence, the applicability of resistance management strategies might vary between species. We discuss potential applications of each of the above strategies to both species. We describe two experiments, one to assess the potential of curative strategy in northern New South Wales and another to assess the potential of the preventive strategy in southern New South Wales. We also describe the process whereby changes have been made in the cotton industry’s strategy to manage resistance to transgenic varieties with incorporation of the use of attract-and-kill.

## Materials and Methods

With highly mobile pests, isolation and/or large treatment areas are necessary to avoid disruption of attract-and-kill experiments by immigrants from nearby untreated areas and to avoid the area-wide impacts on control plots that result from treating the mobile adult stage. Depending on the size of the treated areas, such effects may extend over many km (Mensah et al. 2013). This makes provision of adequate replication difficult because, if control replicates are too close, they will be impacted by nearby applications, but, if they are too far away, they may be too different from the treated areas in, for example, microclimate or local cropping patterns. We minimized this problem by taking multiple samples from different locations in all blocks, before and after treatment.

### Field Site – Curative Trial

**Fig. 1.**
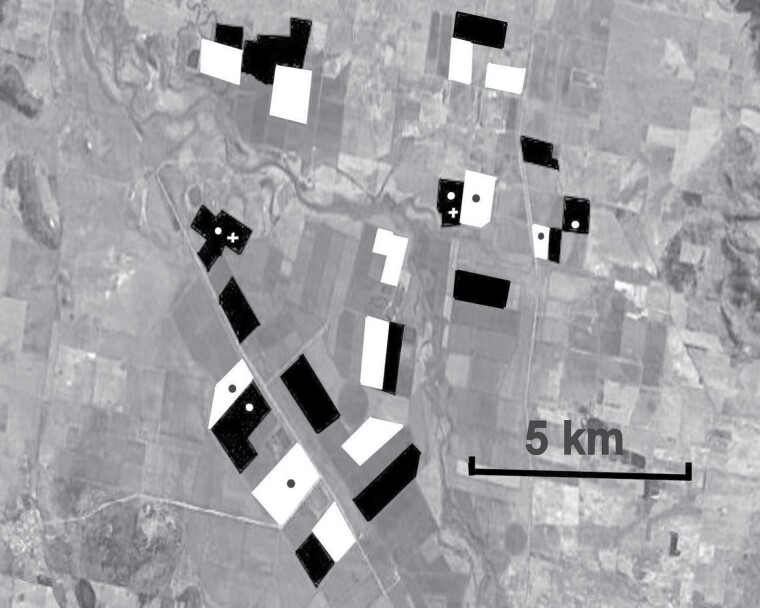
Layout of the curative trial site 20 km SE of Gunnedah, New South Wales, 2012/13. Black areas are blocks (1–3 fields) of cotton treated with three applications of Magnet. White areas are untreated blocks. Each block was associated with a refuge crop of pigeon peas or non-Bt cotton approximately 5% of its area. Circles are light traps, crosses are pheromone traps.

The site (Fig. 1) was located in the Namoi Valley approximately 20 km southeast of Gunnedah, New South Wales (30° 59’S, 150° 15’E). It consisted of 25 blocks of Bollgard II cotton, each containing one to three contiguous fields and ranging from 96 to 360 ha. In the 2012–2013 cotton season, 13 of these blocks were treated with Magnet, and 12 were left untreated. The total area treated was 1,584 ha. Four treated and four untreated blocks (‘local untreated’) were chosen for monitoring sites, and a light trap was installed in each. An additional four light traps were installed in similar untreated cotton blocks approximately 25 km to the south (‘distant untreated’). Pigeon pea refuges (Baker et al. 2016b) totalling approximately 5% of the cotton area were associated with all blocks, but treated areas were chosen to be as far away from them as possible (at least 500 m). Nearby fields were fallow or contained various other crops, including *Helicoverpa* hosts, notably sorghum, *Sorghum bicolor* (L.).

### Field Site – Preventive Trial

**Fig. 2.**
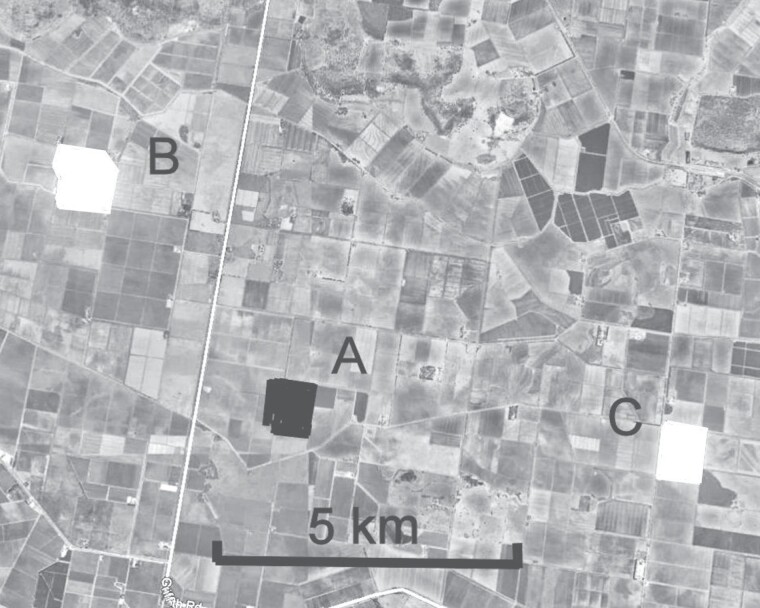
Layout of the preventive trial site 25 km SE of Griffith, New South Wales, 2016/17. Blocks A, B, and C contained one to three contiguous fields of cotton totalling approximately 70 ha for each block, along with a field of approximately 2 ha of pigeon peas grown as a refuge crop. Block A was treated with three Magnet applications. Blocks B and C were untreated.

The site (Fig. 2) was located approximately 25 km SE of Griffith, New South Wales (34° 17’S, 146° 03’E), approximately 520 km southeast of the curative trial site, in the 2016–2017 cotton season. It consisted of blocks of Bollgard 3 cotton containing one to three contiguous fields, all at similar boll-maturing stages, and about six weeks prior to defoliation. Each block was approximately 70 ha and roughly rectangular in shape. Each block also contained approximately 2 ha of pigeon peas. Block A, the treated block, was located between two untreated blocks, at a minimum distance of 5.25 km (untreated Block B) and 6.75 km (untreated Block C). There were no other cotton fields within 5 km of any of the three blocks, and few other host crops for *Helicoverpa* spp. within the area, with nearby fields containing mostly rice, fallow land, or dry pasture.

### Formulation and Application

Magnet was sourced from AgBiTech Australia, Toowoomba, Queensland. The toxicant added to it prior to application was thiodicarb (Larvin 375, Bayer Crop Science, Melbourne), at a rate of 0.5% a.i. The formulation was applied to the entire treated blocks at a rate of 500 ml/100 m of row in bands 1–2 m wide spaced 72 m apart. Application was made using fixed wing aircraft, with techniques similar to those described by Gregg et al. 2016a. In the curative experiment, three applications were made on 6th, 13th, and 19th March 2013. In the preventive experiment three applications, on 13th, 20th, and 25th February 2017 were made.

### Monitoring

In the curative experiment only, light traps were used for monitoring. They consisted of an inverted white fiberglass cone (airport runway markers, with the inside surface polished), placed over a 60 L plastic garbage can, and secured to the ground using bungee cords. The light source consisted of a strip containing 30 waterproof UV LEDS (model SMD 5050, Volka Lighting, Melbourne, Australia) attached to a 30 cm aluminum ring and placed in the bottom of the fibreglass cone. The strip had a power consumption of 3 W, supplied by an 18 Ah sealed lead-acid battery connected to a 20 W solar panel with a 20A MPPT regulator that incorporated a photosensitive switch that turned the light on and off at sunset and sunrise. Insects were collected into a 4 L container of 70% ethanol in the garbage bin underneath the cone. This design ensured the light shone vertically from the traps and could only be seen by insects flying above the trap, and thus would not have attracted insects from a long distance. Light traps were located 50 m into the crop, beside roads running through the center of each of the twelve blocks (four each of treated, local untreated, and distant treated) that were chosen for monitoring (Fig. 1). They were operated on days −9, +5, +15, +22, and +29 relative to the first Magnet application.

In both experiments, each block was monitored using four additional methods: dead moth counts following each application, flush counts for live moths, egg counts, and pheromone trapping. Dead moth counts were performed as described by Del Socorro et al. 2010b in the treated blocks only. Four (curative experiment) or six (preventive experiment) test strips of 50 m long and one meter (one row) wide on the edges of the fields were treated with Magnet at a rate of 250 ml per 50 m using a hand sprayer at the same rate and time of the aerial applications. Test strips on the edges of the fields were used because the dense closed canopy of the crop made finding dead moths along the aerially applied strips within the field very difficult. Bare ground adjacent to the treated rows was searched for dead moths early on the morning following each application and (for the preventive experiment only) on the subsequent three mornings. The toxicant used for the test strips was methomyl (Lannate 225, FMC Australia, North Ryde, New South Wales) at 0.5% a.i. This insecticide kills moths faster than thiodicarb, facilitating their discovery close to treated rows (Del Socorro et al. 2010b). The test strips were searched each morning for 4 d following each application. In the curative experiment, a sample of 23 dead moths was dissected following the first Magnet application to determine species composition, sex, and mated status of the females, as indicated by the presence of spermatophores in the bursa copulatrix. In the preventive experiment following the first application only, a sample of 130 dead moths was similarly dissected. Also following the first application only in both experiments, other noctuid (nontarget) moths were collected and identified.

Flush counts for live moths were performed by walking slowly along 50 m sections of row while throwing soil at the plants ahead, and counting moths which flew off in response to the disturbance. Only moths that resembled *Helicoverpa* spp. in size, color, and flight pattern were counted. In the curative experiment, four flush counts were performed in each of the monitored blocks on days −20, −6, +2, +5, +10, and +17 relative to the first Magnet application. In the preventive experiment, twelve flush counts, spread randomly throughout each block, were performed on days −9, −8, −6, −3, +1, +3, +6, +8, +10, +12, +14, +16, +22, and +25, relative to the first application in each of the three fields. In the curative experiment, four egg counts were performed on the same days as the flush counts in each of the treated, local untreated, and distant untreated blocks at random locations at least 100 m away from the light traps. In the preventive experiment, six egg counts were performed on days −8, −6, −3, 0, +2, +4, +6, +9, +11, +13, +15, +22, and +25 relative to the first application. Egg counts were performed by visual inspection of the plants in six randomly selected one-meter sections. In the preventive experiment a sample of 100 eggs collected from the treated block on days −3, 0, and +2 (i.e., around the first application) was returned to the laboratory and placed in 35 ml plastic cups containing artificial diet (Teakle and Jensen 1991). Surviving pupae were identified to species using morphological criteria (Common 1953).

Pheromone traps were used to obtain moths for carbon isotope analysis rather than to monitor *Helicoverpa* spp. population levels. In the curative experiment, there were two pheromone traps for each species located in treated blocks (Fig. 1) and two for each species in the distant untreated blocks. In the preventive experiment, there was one trap for each species in each block. Universal traps baited with Agrisense pheromones (Entosol Pty. Ltd., Sydney) were used. In the curative experiment, moths collected from the light traps, and, in the preventive experiment, dead moths from the test strips, were also analyzed. Stable carbon isotope ratios were determined using techniques described by Baker and Tann 2013 to ascertain whether they originated from larvae fed on plants with the C3 photosynthetic pathway (including cotton) or from plants with the C4 pathway. In the curative experiment only, *H. armigera* moths were tested for host plant origin because it was considered that *H. punctigera* had no C4 host plants, but, for the preventive experiment, recent evidence (Baker et al. 2019, Bawa et al. 2021) had required a revision of this assumption, so both species were tested.

### Statistical Analysis

Egg and adult flush count data were log_10_ (x + 1) transformed to ensure normality. Analyses of variance with block type and time relative to the first application as factors were performed, followed by one-way ANOVAs of block effects for each day. Where these ANOVAs were significant, they were followed by Fisher’s LSD tests. When normality could not be achieved by log transformation, nonparametric Kruskal–Wallis tests were used. For comparisons of the proportions of moths with C3 and C4 host plant profiles, Χ^2^ tests were used. Minitab Release 16 (Minitab LLC, State College, Pennsylvania) was used for analysis.

## Results

### Curative Experiment

#### Dead Moth Collection

The numbers of dead moths collected after the first application (Table 1) were relatively low compared with moth kills typical of earlier season applications of Magnet (e.g., Del Socorro et al. 2010b). Over 90% were *H. armigera*, and both sexes were present in similar numbers. Half of the females were mated. There were only two non-target moths found. Over the three applications, the total kill from the collection areas was 51 *Helicoverpa* spp., or about 0.13 moths per meter over the eight 50 m test strips for the duration of the trial.

**Table 1.** Numbers of dead moths recovered for one (curative) or four (preventive) days from four (curative experiment, Namoi Valley 012/13) or six (preventive experiment, Griffith, 2016/17) 50 m test strips treated with Magnet, percent females, percent of females mated, and numbers of other noctuid species totalled for all days after the first application only

Application date	H. punctigera			H. armigera			Other noctuid moths
	*N*	% female	% mated	*N*	% female	% mated	
*Curative*							
6 Mar.	2	0	NA	21	53 (19)	50 (10)	2
13 Mar.	0			12			0
19 Mar.	0			16			0
*Preventive*							
13 Feb.	239	58 (110)	48 (64)	25	50 (20)	40 (10)	105
20 Feb.	82			19			
25 Feb.	28			7			

Numbers in parentheses are sample sizes on which the percentages are based.

#### Light Traps

**Fig. 3.**
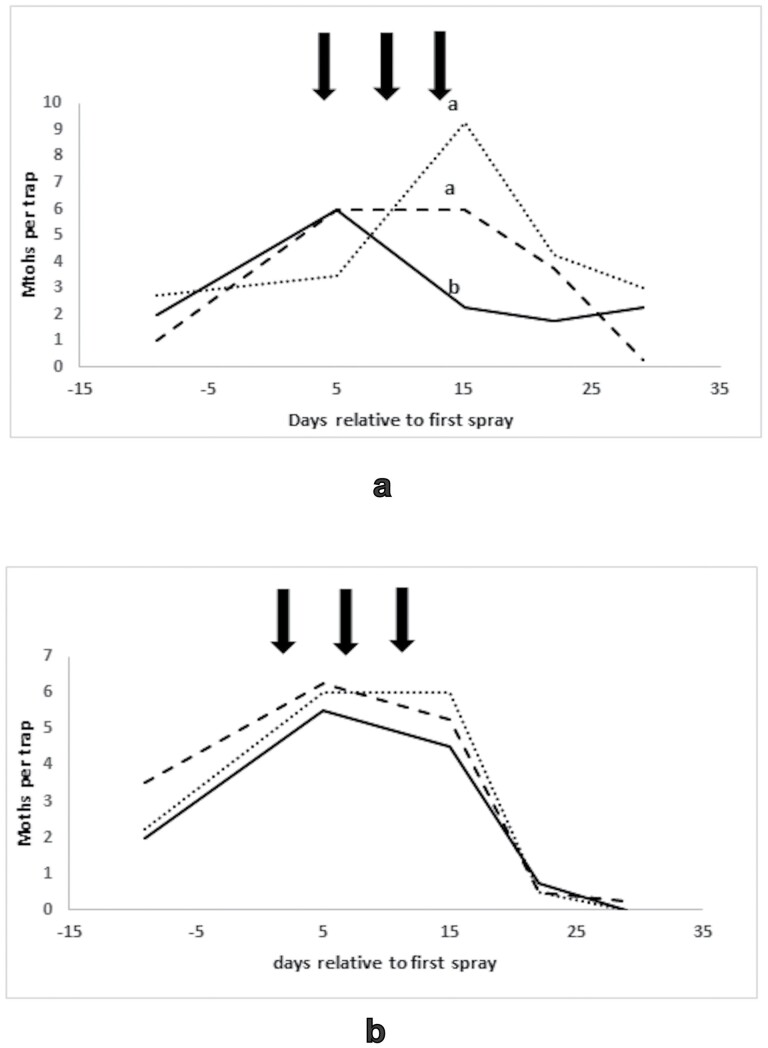
Light trap catches in the curative experiment, Namoi Valley 2012/13, means of eight traps: (a) *H. armigera*, (b) *H. punctigera*. _____ = treated blocks, ------- = local untreated blocks, ……… = distant untreated blocks. Points on the same day bearing different letters are significantly different using Fisher’s LSD test, *P* < 0.05. Arrows indicate the application of Magnet to treated blocks.

Both species were present in similar numbers in light traps for the curative experiment (Fig. 3), in contrast to dominance of *H. armigera* amongst the Magnet-killed moths. There were significant effects of time (F_4,45_ = 3.66, *P* = 0.012 for *H. armigera* and F_4,45_ = 17.71, *P* < 0.001 for *H. punctigera*). Catches were low in the pretreatment phase but rose slightly around the time of treatment before falling to very low levels. For *H. armigera*, there was a trend for lower catches in the treated blocks, which arose mainly from a significant difference on day 15, when numbers were significantly lower in the treated blocks compared to both the local and distant untreated blocks. Most of the *H. armigera* in the pre-treatment phase had carbon isotope profiles indicative of larval origins on C4 plants, which was consistent with the pattern among Magnet-killed moths, but the proportion of moths with the C3 profile increased after the applications, and this increase was statistically significant in the case of the distant untreated blocks (Table 1).

#### Flush and Egg Counts

**Fig. 4.**
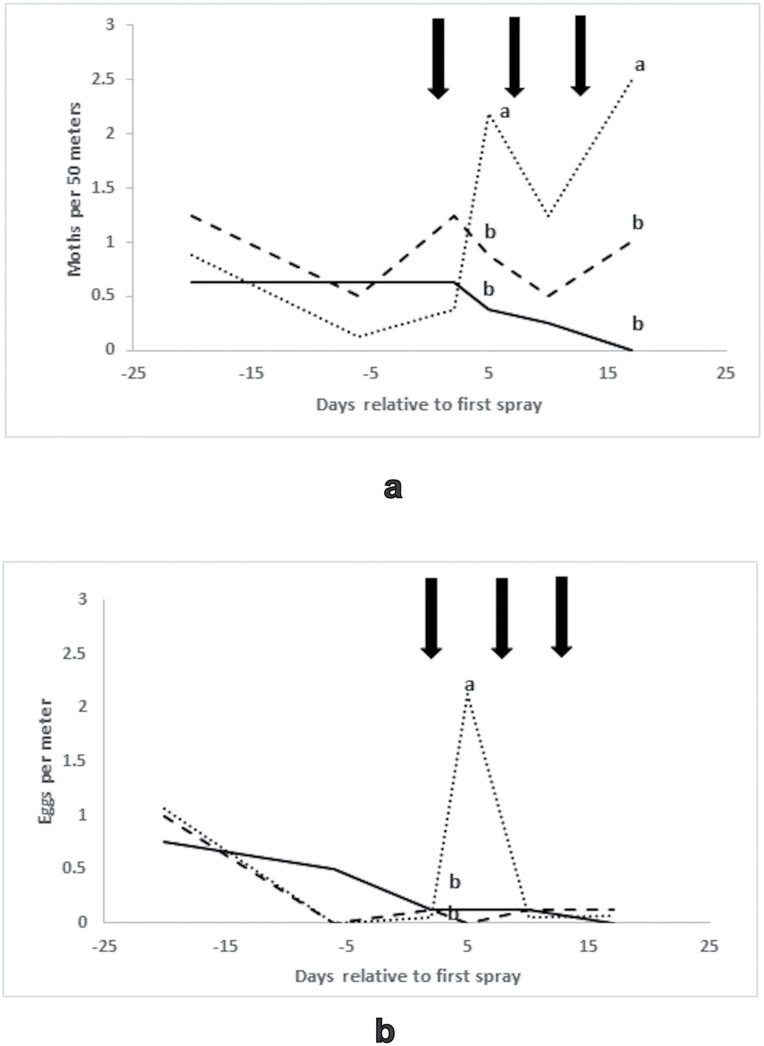
(a) flush counts (moths per 50 m) and (b) egg counts (eggs per meter) from the curative experiment, Namoi valley, 2012/13, means of eight replicates. _____ = treated blocks, ------- = local untreated blocks, ……… = distant untreated blocks. Points on the same day bearing different superscripts are significantly different using Fisher’s LSD test, *P* < 0.05. Arrows indicate the application of Magnet to the treated blocks.

Flush counts for the curative experiment (Fig. 4a) indicated that moth numbers were low compared with those commonly found earlier in the season (P.C. Gregg and A.P. Del Socorro, unpublished data 2005 – 2019). There were significant effects of time (F_5,170_ = 2.55, *P* = 0.029), block type (F_2, 170_ = 9.14, *P* < 0.001), and their interaction (F_10,170_= 4.69, *P* < 0.001). Counts were low in the pretreatment phase in all blocks. They subsequently rose in the distant untreated blocks but not in the treated blocks or the local untreated blocks, leading to statistically significant differences on days 5 and 17, with the distant untreated blocks having higher flush counts than either the treated or local untreated blocks. Egg counts (Fig. 4b) were also low and declining throughout the experiment, with the exception of a significant (Kruskal–Wallis H_2_ = 9.64, *P* = 0.008) but transient increase at day 5 in the distant untreated blocks only, which coincided with increasing flush counts of moths.

#### Carbon Isotope Analysis

Results of carbon isotope analyses of pheromone-trapped *H. armigera* moths (Table 2) were consistent with those from light-trapped moths, indicating a dominance of moths from C4 sources up until treatment began, then a shift to more even proportions of C4 and C3 moths. This shift was statistically significant for moths trapped in the treated blocks.

**Table 2.** Numbers of moths tested for stable carbon isotopes and percentages displaying the C4 host plant profile from various treatments and capture methods in the curative experiment (Namoi Valley, 2012/13) and preventive experiment (Griffith, 2016/17)

			Preapplication		Postapplication	
Area	Catch type	Species	*N*	%C4	*N*	%C4
*Curative experiment*						
Treated	Pheromone	*H. armigera*	99	88^a^	78	32^b^
Distant untreated	Pheromone	*H. armigera*	53	64	162	52
Treated	Light	*H. armigera*	9	89	51	75
Local untreated	Light	*H. armigera*	8	88	76	61
Distant untreated	Light	*H. armigera*	32	80^a^	42	43^**b**^
*Preventive experiment*						
Treated	Pheromone	*H. armigera*	10	100	72	73
Treated	Pheromone	*H. punctigera*	0	NA	50	0
Treated	Magnet kill	*H. armigera*	NA	NA	20	50
Treated	Magnet kill	*H. punctigera*	NA	NA	189	1

Percentages with different superscripts in the same line are significantly different by Χ^2^, *P* < 0.05.

## Preventive Experiment

### Dead Moth Collection

In the test strips after each Magnet application, relatively more moths were killed than in the curative experiment (Table 1). After each application, dead moths were found on all four collection days, indicating that the formulation remained active for at least this period, though there was a tendency for numbers to decline with time. The numbers of moths killed also declined with successive applications. Over the three applications, the total kill from the collection areas was 401 *Helicoverpa* spp., or about 1.36 moths per meter over the six 50 m test strips for the duration of the trial.

*H. punctigera* were more abundant than *H. armigera* among the dead moths, representing 90% of the kill following the first application, 81% following the second, and 80% following the third. These proportions were significantly different between the three applications (Χ^2^_2_ = 7.54, *P* = 0.02). Following the first application, approximately equal numbers of male and female moths of both species were killed, and the proportion of mated females was slightly less than 50%.

Nontarget moths were only collected following the first application and accounted for approximately 28% of the total kill. Of the 105 specimens collected, there were 56 *Spodoptera exigua* (Hübner), 19 *Agrotis ipsilon* (Hufnagel), 14 *Mythimna loreyimima* (Rungs), seven *Mythimna convecta* (Walker), four *Agrotis munda* (Walker), one *Cosmodes elegans* (Donovan), and five specimens which could not be identified.

### Flush and Egg Counts

**Fig. 5.**
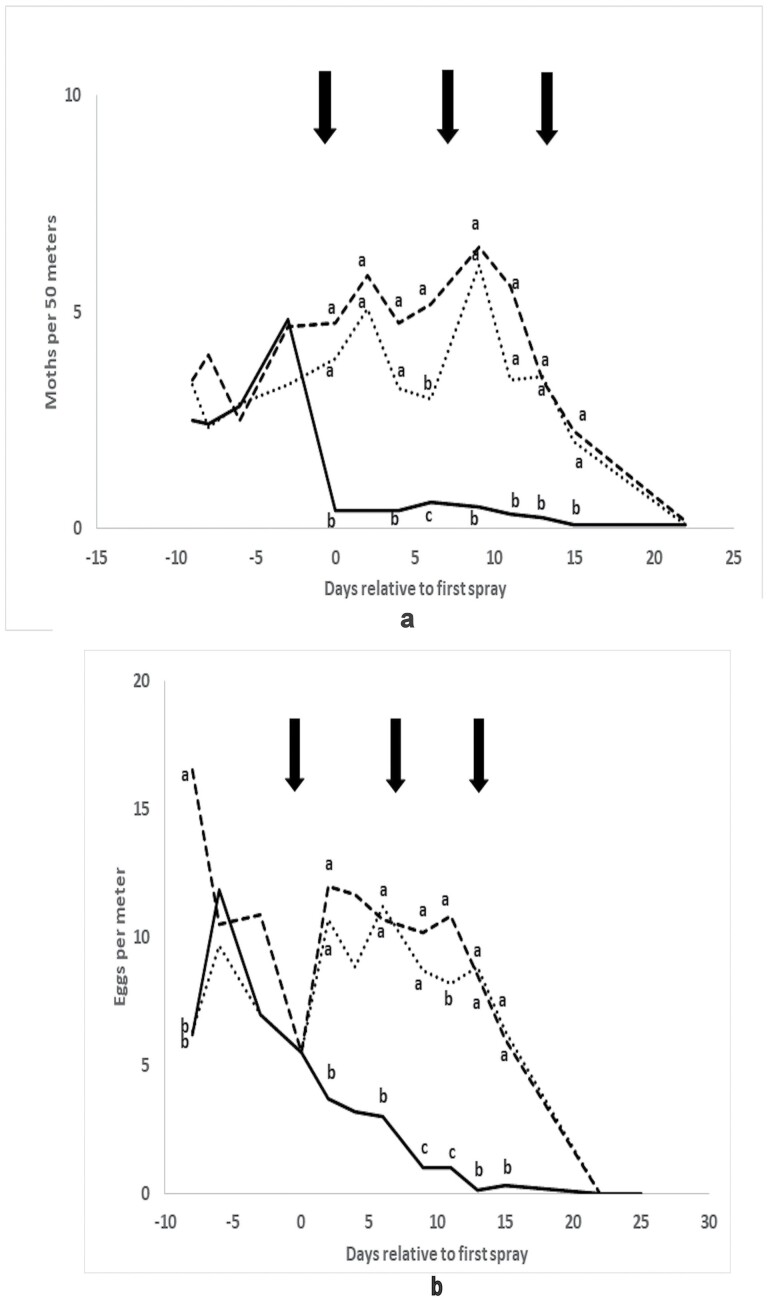
(a) flush counts (moths per 50 m, means of 12 replicates) and (b) egg counts (eggs per meter, means of six replicates) from the preventive experiment, Griffith 2016/17. _____ = Treated Block A, ------- = untreated Block B, ……… = untreated Block C. Points on the same day bearing different superscripts are significantly different using Fisher’s LSD test, *P* < 0.05. Arrows indicate the application of Magnet to the treated Block A.

Both flush and egg counts (Figs. 5a and b) were substantially higher than for the curative experiment. For the flush counts, an ANOVA model accounted for 64.4% of the variance, and the effects of block (F_2, 165_ = 93.6, *P* < 0.001), time (F_10, 165_ = 8.9, *P* < 0.001), and the interaction (F_20, 165_ = 5.5, *P* < 0.001) were all significant. One-way analyses of variance indicated no significant differences between the blocks prior to the first application, but, after then, there were significant differences (*P* < 0.001) between the blocks on all days except day 25. These differences were mainly due to lower moth numbers in the treated block compared with the two untreated blocks. There was a tendency for untreated Block B to have higher numbers than untreated Block A throughout the experiment, but the difference was significant only on day 6 after the first application. Compared with the combined means of the two untreated blocks, numbers of moths in the treated block were reduced by between 90% (4 d after the first application) and 93% (2 d after the third application).

On days 22 and 25 after the first application, all egg counts on all three blocks were zero. If these two days were omitted an ANOVA model accounted for 69.5% of the variance, and the effects of block (F_2, 498_ = 232.8, *P* < 0.001), time (F_13, 498_ = 40.3, *P* < 0.001), and the interaction (F_26, 498_ = 11.1, *P* < 0.001) were all significant. One-way analyses of variance followed by Fisher’s LSD tests showed that the only occasion prior to treatment, when there were significant differences between blocks, was 8 d preapplication, when untreated Block B had significantly higher numbers than the other two (Fig. 3). On every occasion after the application, except when egg numbers had fallen to zero across all blocks, the treated Block A had significantly lower egg numbers than either of the untreated blocks. Compared with the combined means of the two untreated blocks, numbers of eggs in the treated block were reduced by between 78% (2 d after the first application) and 95% (3 d after the third application). Of the sample of eggs collected from the treated field around the time of the first application, 39 survived to the pupal stage, and, of these, 33 (85%) were *H. punctigera* with the remainder being *H. armigera.*

### Carbon Isotope Analyses

Among 189 dead *H. punctigera* moths collected from the test strips, 187 (98.9%) had carbon isotope ratios indicative of feeding on plants with the C3 pathway, which includes cotton and pigeon peas, while only two had ratios indicative of feeding on C4 plants (Table 2). For *H. armigera*, among 20 dead moths collected from the test strips, 50% had C4 profiles. The low numbers of dead *H. armigera* moths were supplemented by 72 moths collected from the pheromone trap in the treated block. Of these, 20 (27%) had isotope ratios indicative of feeding on C3 plants, and the remaining 52 (73%) had ratios indicative of feeding on C4 plants.

## Discussion

### Impacts of Bisexual Attract-and-Kill

The numbers of dead moths collected from the test strips indicated that substantial moth kills were obtained from each of the three applications, especially in the preventive experiment. The numbers killed declined progressively with each application, mostly because the numbers present (as judged by flush counts; Figs. 4a and 5a) were lower for the second and third applications. Flush counts do not result in accurate identifications of every moth, because they are made on moths in flight at a distance of up to several meters. However, the species composition of the dead moth collections suggested that flush counts mainly recorded *Helicoverpa* spp. with only minor errors possible due to species that could be mistaken for *Helicoverpa* spp. (notably *Mythimna* spp. and *Agrotis munda* in the preventive experiment). For the curative experiment, total numbers of dead *Helicoverpa* spp. averaged 0.13 moths per meter across the eight test strips. Extrapolating this to the total of 221.9 × 10^3^ m of aerially applied treatment band gives a total kill of 28.8 × 10^3^. For the preventive experiment, the corresponding estimates are an average of 1.36 per meter across all six test strips, and a total of 13.4 × 10^3^ m of treatment band, giving a total kill of 18.2 × 10^3^ moths. These figures are likely to be considerable underestimates because searching adjacent furrows along treated rows recovers less than 10% of the moths that are killed (Del Socorro et al. 2010b). Both male and female moths were killed, including both mated and virgin females.

Flush counts suggested that the numbers of moths in treated blocks were immediately reduced after the first application, by at least 50% for the curative experiment and by about 90% for the preventive experiment. This reduction was maintained through successive applications for about 20 d, until moth numbers fell in most blocks. The most likely explanation for this is that most of the resident moth population was killed by the first applications and subsequent applications killed immigrants or locally emerging moths as they arrived. These patterns were repeated, after a short delay, in egg numbers in the preventive experiment. In the absence of density-dependent mortality, the reduction in oviposition represented a reduction of approximately 90% in the pool of larvae that would be exposed to selection for Bt resistance, and in the numbers of overwintering pupae that would otherwise require pupae busting. It is unlikely that density-dependent mortality would have been significant because the density of eggs was low, and mortality of early instars on Bt cotton is usually greater than 99% (Bahar et al. 2019). This low density of immature stages made confirmation of the efficacy of the treatment at the larval or pupal level impractical, but it is clear that the applications substantially reduced the opportunity for selection for resistance. In the curative experiment, egg numbers were very low throughout, except for a transient increase in the distant untreated blocks. The decrease in moth and egg numbers at the end of the monitoring period, in both experiments, was probably due to declining attractiveness and suitability for larval survival of the crop as it dried out following the last irrigation, which occurred around the time of the first Magnet application for the curative experiment and the third application for the preventive experiment. The decline in host quality was especially marked in the curative experiment, where most crops were defoliated around the time of the third application, and it is unlikely that any eggs laid from the time of the first application would have survived to pupation.

In the curative experiment, trends in flush and egg counts in the local untreated blocks in general resembled those of the nearby treated blocks, rather than the distant untreated blocks. While the possibility of different sources of moths arriving at the distant untreated blocks compared with the treated and local untreated blocks cannot be excluded, these trends are consistent with area-wide impacts resulting from treatment of large areas with attract-and-kill products (Mensah et al. 2013).

Results from the light traps and dead moth collections in the curative experiment indicated that the dominant species of *Helicoverpa* at the time of first application was *H. armigera*. In contrast, for the preventive experiment, the results from dead moth collections and laboratory rearing of eggs suggested that the dominant species of *Helicoverpa* (>80%) was *H. punctigera*. The latter finding is not in agreement with studies from the pre-Bt GM cotton era, which indicated that *H. armigera* was then the dominant species in southern New South Wales in late summer (Duffield and Steer 2006). Further work is required to determine whether the contrast between our data showing late-season dominance of *H. punctigera* and the earlier data of Duffield and Steer 2006 showing dominance of *H. armi*gera at this time represents a long-term, seasonal trend similar to that described for northern New South Wales by Baker and Tann 2017.

Carbon isotope analyses indicated that, although there was a trend for increasing proportions of moths from C3 crops as both experiments progressed, most of the *H. armigera* that were killed by the applications originated from C4 crops. The most likely source was sorghum, which was common in the general area of both sites. In contrast, almost all *H. punctigera* moths originated from C3 plants, which might include cotton and its associated pigeon pea refuges, but might also include noncrop species (Gregg et al. 2019). This is consistent with known host preferences for *H. punctigera* (Cunningham and Zalucki 2014). Larvae of *H. punctigera* do not survive on sorghum, and, although *H. punctigera* moths with the C4 host profile are sometimes found in cropping regions (Baker et al. 2019) and there are noncrop C4 hosts that can support larvae (Bawa et al. 2021), there were almost no *H. punctigera* with the C4 profile in these experiments.

### Strategies for Using Bisexual Attract-and-Kill Technologies in Resistance Management

In both of these experiments, substantial numbers of *Helicoverpa* spp. moths were killed. However, for resistance management, it is not sufficient to merely kill moths. It is necessary to disproportionately kill the right kind of moths – those which have a high frequency of resistance alleles because they have been exposed to selection pressure as larvae, or those whose progeny are likely to be thus exposed. Of the three potential strategies for using bisexual attract-and-kill technologies in resistance management outlined in the Introduction, it is likely that the preventive approach, exemplified by the second experiment in this study, best meets these criteria. It is therefore likely to be the most effective alternative to pupae busting in those regions where successfully undertaking the mandatory cultivation can be impacted by wet winters. There is little evidence that the curative and remedial approaches target the most appropriate moth cohort. The curative approach, exemplified by the first experiment reported here, killed moths that had carbon isotope profiles reflective of the regional and seasonal crop mix rather than cotton. Unpublished data (S. Downes and P.C. Gregg, 2012–2015) indicates that moths collected from pheromone traps in the Namoi Valley at the end of the season do not have a higher frequency of resistance to Cry2Ab than moths at other times in the season. Combined with the low numbers of moths found in the curative experiment reported here, this indicates that there is little evidence of a potential target cohort of late-season moths emerging from cotton.

The remedial approach has also been investigated (P.C. Gregg and A.P. Del Socorro, unpublished data 2010 and 2016) using Magnet applications on spring wheat to target overwintering moths as they emerge. Almost all the moths killed were *H. punctigera* and were probably immigrants from distant, noncrop sources where there would be no selection for resistance. It is thus unlikely that either the curative or remedial approaches would contribute as much to the management of regional resistance frequencies as the current requirement for remediating ineffective or no pupae busting, which is increasing refuge area in the next season.

In contrast, the preventive approach, exemplified by the second experiment reported here targets the cohort of moths which lays the last generation of eggs likely to reach maturity before the crop is defoliated. This has the advantage of directly reducing the numbers of larvae which would otherwise be subjected to selection pressure and require pupae busting. While this could be done using insecticides targeting the larvae (Lu et al. 2012), Magnet has the advantage of being a more cost-effective option (with product being applied to 1 row in 72 as opposed to a broadcast over-the-top larvicidal insecticide application). It is also more efficient with regard to application costs and time and minimizes impact on natural enemies of *Helicoverpa* spp. or other late-season pests (Gregg et al. 2016b).

### Implementation of Bisexual Attract and Kill Products in the Transgenic Resistance Management Strategy

The use of transgenic cotton varieties expressing insecticidal toxins in Australia is controlled by the Australian Pesticides and Veterinary Medicines Authority (APVMA). For Bollgard cotton, the Authority has required the development of a comprehensive resistance management strategy implemented by the technology providers through a Resistance Management Plan that is supported by the cotton industry. Industry input is provided by the Transgenic Insecticide Management Strategy committee of Cotton Australia (Cotton Australia 2021) which comprises regional cotton industry representatives along with representatives of major government research organizations and related industries such as grains. The Transgenic Insecticide Management Strategy (TIMS) committee is advised by a Transgenic (Bt) Technical Panel, with a majority of independent scientists. This panel meets annually, at the end of each cotton season, to review data on any changes in resistance frequency and information on levels of compliance with requirements of the Resistance Management Plan, supplied by technology providers. The panel also reviews, in collaboration with technology providers, new research on current and emerging tactics for resistance management. The panel along with the technology provider advises the TIMS committee of any changes to the Resistance Management Plan that might be appropriate. The committee is required to approve any recommended changes to the plan prior to approval being sought from the Australian Pesticides and Veterinary Medicines Authority. In meetings following the 2017 and 2018 seasons, the Bt Technical Panel together with Bayer considered evidence relating to the use of bisexual attract-and-kill technology, including data in this paper. It recommended the addition of the strategy as an optional alternative to pupae busting in circumstances where the latter would otherwise be required.

After ratification by the Bt Technical panel and the technology provider, the changes were approved by the Australian Pesticides and Veterinary Medicines Authority for inclusion in the 2020-2021 Resistance Management Plan (Cotton Australia 2021). These changes required growers to opt in to apply the attract and kill strategy at planting, but allowed them to opt out up until 1 February. Growers remaining in the strategy were required to deliver three weekly applications, commencing no earlier than February 10 with the final application being no later than March 1. These regulations were applied to the southernmost cotton growing regions (the Lachlan, Murrumbidgee, Menindee, and Murray areas), but growers in more northerly regions could apply to the technology provider for special permission to use the strategy.

In 2019 and 2020, farm-scale treatments were conducted to test the effectiveness and commercial viability of using the bisexual attract-and-kill alternative to pupae busting. In 2021, after the successful conclusion of the two pilot years and approval of the addition to the Resistance Management Plan by the Australian Pesticides and Veterinary Medicines Authority, a commercial offering and associated documentation system was devised and executed covering 21,520 ha of cotton treated three times. This involved 489 individual fields managed by 110 separate grower entities and represented approximately 60% uptake of the possible area that would not be defoliated prior to the end of March. Fifteen aerial operators were trained and accredited in aerial application of the product. No major compliance issues were noted. While pupae busting is still the preferred end of season strategy for resistance management for Bt cotton, it is likely that attract-and-kill technologies may become an increasingly used option for end-of-season resistance management for Bt cotton in southern Australia.
